# Host stage and temperature for the rearing of *Aridelus rufotestaceus* (Hymenoptera: Braconidae), with notes on acceptance and suitability of 2 stink bug species

**DOI:** 10.1093/jisesa/iead062

**Published:** 2023-09-18

**Authors:** Santolo Francati, Antonio Martini, Maria Luisa Dindo

**Affiliations:** Department of Agricultural and Food Sciences (DISTAL), Alma Mater Studiorum Università di Bologna, Viale Fanin 42, 40127 Bologna, Italy; Department of Agricultural and Food Sciences (DISTAL), Alma Mater Studiorum Università di Bologna, Viale Fanin 42, 40127 Bologna, Italy; Department of Agricultural and Food Sciences (DISTAL), Alma Mater Studiorum Università di Bologna, Viale Fanin 42, 40127 Bologna, Italy

**Keywords:** parasitoid, rearing, temperature, host stage

## Abstract

*Nezara viridula* (L.) (Hemiptera: Pentatomidae) is a harmful pest of many agricultural crops in different parts of the world. This stink bug is the preferred host species of *Aridelus rufotestaceus* Tobias (Hymenoptera: Braconidae), an endoparasitoid of nymphs and adults of pentatomids. With the aim to improve the rearing procedure of this beneficial insect, the acceptance and suitability of all mobile stages of *N. viridula* (from first instar nymph to adult) were evaluated. At 25 °C, all host stages were accepted and suitable for *A. rufotestaceus* development, but the highest parasitoid cocoon and adult yields were obtained from second instar nymphs. The possibility to reduce the development time of *A. rufotestasceus* by increasing the rearing temperature was also evaluated, but 28 °C proved to be detrimental for parasitoid development, as shown by the very low cocoon and adult numbers obtained. The acceptance and suitability of the invasive pentatomid species *Halyomorpha halys* (Stål) for *A. rufotestaceus* was also tested. Female wasps were observed piercing *H. halys* nymphs with the ovipositor, but no cocoons were obtained, nor were larvae or head capsules detected in the exposed stink bugs.

## Introduction

*Nezara viridula* (L.) (Hemiptera: Pentatomidae), the southern green stink bug, is a harmful pest of many agricultural crops in different parts of the world (Todd 1989, Panizzi et al. 2000, Kiritani 2011). Originated from the Afrotropical region (Jones 1988, Kavar et al. 2006), it is now considered a cosmopolitan species (Panizzi et al. 2000, Esquivel et al. 2018). Over 50 parasitoid species attacking *N. viridula* have been identified, most of which were egg parasitoid wasps (Jones 1988, Esquivel et al. 2018). The other species were mainly dipteran tachinids of the subfamily Phasiinae. These flies usually attack adults, but they are also capable of parasitizing late-instar nymphs (Jones 1988, Francati et al. 2019). The only known hymenopterous parasitoid recorded to be capable of parasitizing both nymphs and adults is *Aridelus rufotestaceus* Tobias (Hymenoptera: Braconidae), a solitary thelytokous koinobiont endoparasitoid, which pupates outside the host in silk cocoons (Shaw et al. 2001, Ruberson et al. 2009). This species is probably native to the Sino-Russian region, but it has also been recorded in Italy (Shaw et al. 2001), the United States (Ruberson et al. 2008), Madeira (van Achterberg and Aguiar 2009), Korea (Lee et al. 2017), New Zealand (Martin 2017), Malta (van Achterberg 2022) and, recently, Japan (Fujie and Maeto 2022). Under field conditions, *A. rufotestaceus* was found to emerge from other pentatomid species besides *N. viridula*, including *Glaucias amyoti* (Dallas) in New Zealand, *G. subpunctatus* (Walker) in Japan, and *Euschistus servus* (Say) in the United States (Ruberson et al. 2009, Martin 2017, Fujie and Maeto 2022). Under laboratory conditions, *E. quadrator* Rolston, *Chinavia hilaris* Say (=*Acrosternum hilaris* Say), and *Piezodorus guildinii* (Westwood) were also successfully parasitized (Ruberson et al. 2009).

Although *N. viridula* is the most recorded host of *A. rufotestatceus* (Aduba et al. 2013), in the field the rates of parasitism were low, ranging from less than 5% in the Umbria region (center of Italy) and Georgia (USA) to 21.7% in Lazio region (center of Italy) (Shaw et al. 2001, Ruberson et al. 2009). These low rates were possibly related to a recent introduction of *A. rufotestaceus* in the study areas (Shaw et al. 2001, Ruberson et al. 2009). Under laboratory conditions, Shaw et al. (2001) found rates of parasitism of 95% and 85.7%, respectively, in third and second instar *N. viridula* nymphs, but did not report the number of individuals exposed to *A. rufotestaceus.*

The parasitoid development time was 40 days on average at 25 ± 1 °C and about 63 days at 20 ± 1 °C (Shaw et al. 2001, Ruberson et al. 2009). At 30 ± 1 °C, *A. rufotestaceus* development was faster, about 36 days, but mortality dramatically increased, since only 1 adult was obtained from *N. viridula* second instar nymphs (vs. 18 adults emerged at 25 ± 1 °C) (Ruberson et al. 2009).

In this study, we assessed the suitability of all mobile stages of *N. viridula* (including first instar nymphs, never tested before), as well as the influence of temperature on parasitoid development. Moreover, the acceptance and suitability of *Halyomorpha halys* (Stål) was tested. Although this invasive pentatomid pest is native to the same areas of *A. rufotestaceus* (Cianferoni et al. 2018), it has not yet been recorded as a host for *A. rufotestaceus*. For both pentatomid species, suitability was also investigated through anatomical observations. The objective of this research was to increase knowledge on *A. rufotestaceus* biology, rearing procedures, and host range, with the ultimate goal of evaluating the possibility to mass produce this parasitoid for use in biological control programs against the target insect pests.

## Materials and Methods

### Insects

Colonies of *N. viridula* and *H. halys* were established in 2015 and periodically augmented with adults collected in the Bologna area (44°29ʹ38″N 11°20ʹ34″E) (Emilia Romagna region, northern Italy). *Nezara viridula* was fed on celery (*Apium graveolens* L.) and seeds of soybean (*Glycine max* [L.] Merrill) and sunflower (*Helianthus annuus* L.) (Salerno et al. 2006). Besides soybean and sunflower seeds, *H. halys* was fed on green beans (*Phaseolus vulgaris* L.), carrots (*Daucus carota* L.), and kiwifruit (*Actinidia chinensis* Planch.) (Dingha and Jackai 2016). Adults of both species were kept in plastic boxes (24 ʹ 16 × 36 cm) with a squared hole (6 × 10 cm) closed with a fine metallic mesh on their lids. Each box contained 30–40 adults. Oviposition occurred on paper sheets placed in the boxes. Egg masses were collected twice a week and transferred to 6-cm diameter Petri dishes with a green bean and a cotton swab soaked in tap water (1 × 3 clusters per dish). After hatching, which under laboratory conditions occurred after about 6 days, nymphs were maintained in the same dishes until the second instar, when they were moved to 18 × 10 × 36 cm plastic boxes. They were fed as described above for adults until emergence.

The colony of *A. rufotestaceus* was started with adults emerging from *N. viridula* nymphs that were field collected at Faenza (44°17ʹN 11°53ʹE) (Emilia Romagna region, northern Italy) in early fall 2018. The parasitoid species was identified following Shaw et al. (2001) and using dichotomous keys (Muesebeck 1936, Chen and van Achterberg 1997, Lee et al. 2017). Voucher specimens were deposited at the Department of Agricultural and Food Sciences of the University of Bologna. The parasitoid adults (10–15) were kept in a plexiglass cage (20 × 20 × 20 cm) and fed on cotton balls soaked in a sugar and distilled water solution (10% sugar). Parasitization occurred by exposing *N. viridula* third instar nymphs (Shaw et al. 2001) to *A. rufotestaceus* females (40 nymphs/4 females) for 2 h on a weekly basis. After exposure, the nymphs were moved to 18 × 10 × 36 cm plastic boxes and fed as described above. When parasitoid cocoons formed (about 20 days after exposure), they were transferred to a plexiglass cage (described above) for emergence.

Insect rearing and laboratory tests were conducted under controlled conditions (25 ± 1 °C, 70 ± 5% RH. and 16:8 L:D photoperiod), if not indicated differently.

### Host Stage

The experiment was conducted to determine the most suitable *N. viridula* stage for the development of *A. rufotestaceus* under no-choice conditions. Egg masses of *N. viridula*, laid within 24 h, were placed singly in 6-cm diameter Petri dishes and maintained as described above. Following the method described by Dindo et al. (2016) for another braconid (*Dinocampus coccinellae* [Schrank], a parasitoid of ladybugs), stink bugs were exposed to wasps in plexiglass cylinders (20 cm high, 9 cm diameter). All host mobile stages (i.e., 5 nymphal stages [N1, N2, N3, N4, N5] and the adult) were tested. They were identified following Panizzi (2004). For each stage, 5 individuals were placed in a cylinder with one 1–2-day old parasitoid female for 1 h. First instars were exposed within 30–36 h after egg hatching to allow nymphs to assimilate the symbiotic bacteria left by females on eggs, which are essential for the growth, development, and survival of *N. viridula* and *H. halys* (Tada et al. 2011, Taylor et al. 2014). For the other stages, newly-molted individuals were utilized. After exposure to wasps, the stink bugs of each stage were removed from cylinders, placed in plastic boxes (18 × 10 × 36 cm), and monitored daily for 30 days to detect the number of newly-formed parasitoid cocoons (i.e., 10 days more than the average time from host exposure to cocoon detection, observed in the standard colony). The cocoons were placed individually in plexiglass cylinders (10 cm high, 9 cm diameter) and checked daily until adult emergence. For each stage, 5 individuals were left unexposed and maintained as controls. After 30 days, the number of surviving stink bugs was counted for both exposed and unexposed stink bugs.

Five replicates per stage, each corresponding to a group of 5 exposed and 5 unexposed individuals were performed. An overall number of 25 exposed and 25 unexposed individuals per stage was thus utilized for a total of 300 individuals.

To evaluate the results, the number and percentage of hosts which produced a parasitoid cocoon (=parasitoid cocoon yield) were calculated. The number and percentage of hosts which produced a parasitoid adult (=parasitoid adult yield) and the parasitoid development times (in days) from egg to cocoon (i) and total (from egg to adult) (ii) were measured. The Degree of Infestation (DI), measuring the proportion of hosts that were killed by the parasitoid, was estimated as (*T* − di)/*T*, where *T* = number of survived individuals in unexposed (control) stink bugs and di = number of survived stink bugs in exposed ones (adapted from Chabert et al. 2012).

### Influence of Temperature

This experiment was conducted to determine if an increase in *A. rufotestaceus* rearing temperature resulted in a concomitant reduction in development time. At 25 ± 1 °C (the standard temperature maintained in our colony) the parasitoid takes about 40 days to reach adulthood (Ruberson et al. 2009). In this experiment, the selected temperature was 28 ± 1 °C, in between the standard temperature and the temperature tested by Ruberson et al. (2009) (30 °C), which proved to be detrimental. Following the results achieved in the experiment described above, newly-molted second instar *N. viridula* nymphs were placed in 2 cylinders (20 cm height, 9 cm diameter) and exposed for 1 h to parasitoid females (5 nymphs/1 female). Exposure occurred at 25 ± 1 °C. Subsequently, each group of nymphs was removed from the cylinder and transferred to a plastic box (18 × 10 × 36 cm). The 2 boxes with nymphs were maintained either at 28 ± 1 °C or at 25 ± 1 °C (controls). A control group of 5 unexposed nymphs was also kept for each temperature. Five replicates were performed, with an overall number of 25 nymphs per treatment, for a total of 100 nymphs. All stink bugs were monitored daily for 30 days to detect dead hosts and newly-formed parasitoid cocoons.

The hosts which produced a parasitoid cocoon and a parasitoid adult were counted. The development times were measured as described in the “Host stage” experiment. The dead stink bugs (either exposed or unexposed to parasitoid) were also counted.

### Acceptance and Suitability of *H. halys*

Acceptance and suitability of the nonhost species *H. halys* for *A. rufotestaceus* was tested by exposing second instar nymphs of *H. halys* to *A. rufotestaceus* (5 nymphs/1 parasitoid female in a cylinder). Second instar was selected for both stink bugs, because it produced the highest cocoon and adult yields in the “Host stage” experiment for *N. viridula.* Unexposed nymphs (5) were maintained as controls. Five second instar *N. viridula* nymphs were also exposed to parasitoid to make a comparison between the 2 pentatomids in terms of parasitization success and mortality occurrence. Following exposure, all nymphs were kept under the same conditions described in the “Host stage” experiment and monitored daily for 30 days. Also in this experiment, 5 replicates were carried out for a total of 50 *H. halys* and 50 *N. viridula* (for each species, 25 exposed and 25 unexposed nymphs).

For each species, the exposed hosts which produced a parasitoid cocoon (=parasitoid cocoon number), and a parasitoid adult (=parasitoid adult number) were counted, as well as dead stink bugs (exposed or unexposed to a parasitoid).

### Statistical Analysis

In the “Host stage” experiment, the parasitoid cocoon and adult yields, the DI, and the development times were analyzed by a nonparametric median test, because heteroscedasticity occurred (Levene’s test) and data were not normally distributed (Shapiro–Wilk test) (Zar 2010). As the data for the development times were not available for all replicates, they were pooled for the 5 replicates. The median test was followed by a nonparametric multiple comparison when a significant difference occurred (Dunn’s test, *P* = 0.05).

In the “Influence of temperature” experiment, the number of parasitoid cocoons, adults, and dead stink bugs were analyzed by Log-linear analysis (Marginal association test). Independent variables were temperature (25 vs. 28 °C) and replicates (5). The development times were pooled for the 5 replicates and were analyzed by a nonparametric median test, because heteroscedasticity occurred (Levene’s test) and data were not normally distributed (Shapiro–Wilk test) (Zar 2010). In the “Acceptance and suitability of *H. halys*” test, the numbers of dead stink bugs in exposed and unexposed hosts were analyzed by Log-linear analysis, and independent variables were host species (*N. viridula* vs. *H. halys*) and replicates (5). For *H. halys*, the Log-linear analysis was used to ascertain whether or not exposure to parasitoid influenced the number of dead stinkbugs. The number of parasitoid cocoons and adults and the development times were not analyzed, because no parasitoid was obtained from *H. halys.*

Statistical tests were done using STATISTICA 10.0 (Statsoft Inc. 2010).

### Anatomical Studies

Anatomical studies were conducted to detect the presence or absence of *A. rufotestacues* and determine the developmental state of the parasitoid in *N. viridula* or *H. halys*. For both stink bugs, 5 third instar nymphs were exposed (separately for each species) to 1 *A. rufotestaceus* female for 1 h, following the methods described for the “Host stage” experiment. Third instar was selected as the host stage in lieu of the second, because of its bigger size, which could facilitate anatomical studies. An overall number of 40 individuals per species were exposed. After exposure, the nymphs were maintained as in the standard parasitoid colony and checked daily until death. Rotting specimens, which looked unsuitable for dissection, were discarded. The selected dead specimens were stored at −18 °C, then dissected in saline (Ringer’s) solution and finally examined under a stereomicroscope. The presence and extent of injuries to internal organs, melanization (indicating the host’s immune reaction), and presence of parasitoid larvae, and their instar were assessed. To highlight the host’s sclerified parts, samples were cleared in KOH saturated solution for 24 h at room temperature, then placed in distilled water for another 24 h. Finally, they were mounted in permanent slides with Faure’s medium.

## Results

### Host Stage

**Fig. 1.**
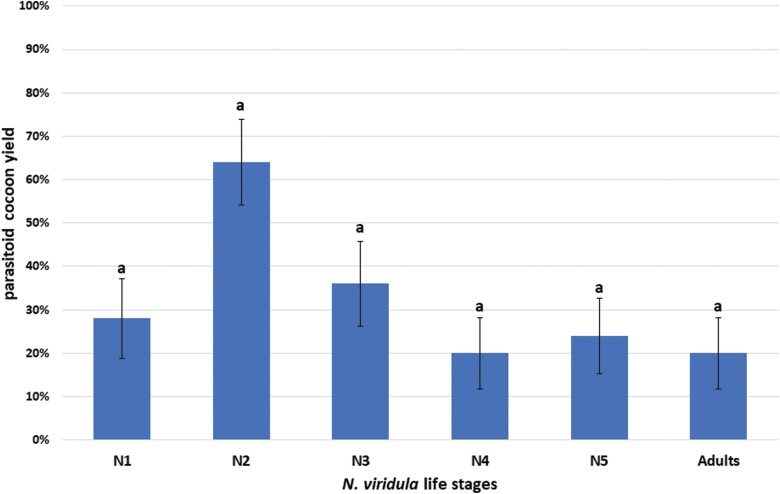
Effects of host stage at parasitization on the parasitoid development in the system *Nezara viridula*–*Aridelus rufotestaceus*: hosts which produced a parasitoid cocoon (=parasitoid cocoon yields) (%). The columns indicate the mean values ± SE. Number of replicates = 5 per stage, each comprising 5 individuals. Different letters above the columns indicate differences in the percentages, as determined by Median test followed by Dunn’s test.

Cocoons of *A. rufotestaceus* were obtained from all stages of *N. viridula*. Cocoon yield was higher in N2 compared to N1, N3, N4, N5, and adults. The difference was close to 0.05 significance level (χ^2^ = 10; df = 5; *P* = 0.075, median test) (Fig. 1). The adult yields were 24.00 ± 14.69 (N1), 56.00 ± 13.27 (N2), 36.00 ± 22.27 (N3), 20.00 ± 6.32 (N4 and N5), 20.00 ± 15.49 (adult). This parameter was not significantly influenced by host stage at parasitization (χ^2^ = 6.56; df = 5; *P* = 0.252, median test).

Median test was significant for the parasitoid development times from egg to cocoon (χ^2^ = 20.37; df = 5; *P* = 0.011) and from egg to adult (χ^2^ = 13.09; df = 5; *P* = 0.005). The total development time from egg to adult ranged from 42 to 45.5 days, and the shortest times were observed in *N. viridula* third instar nymphs (Table 1).

**Table 1.** Effects of host stage at parasitization on the parasitoid development in the system *Nezara viridula*–*Aridelus rufotestaceus*: parasitoid development times in days from egg to cocoon and from egg to adult. For each time, the number of replicates (=number of individuals) is indicated in brackets to the left of the means (±SE). Means within a column followed by the same letters are not significantly different (median test followed by Dunn’s multiple comparison test)

Host stage	Parasitoid development time (in days)	
	From egg to cocoon	From egg to adult
N1	(7) 25.14 ± 0.67a	(6) 45.5 ± 0.67a
N2	(16)23.5 ± 0.79a	(14) 44.93 ± 1.03ab
N3	(9) 21.33 ± 1.21b	(9) 42 ± 0.78b
N4	(5) 22 ± 0.71ab	(5) 43.2 ± 0.49ab
N5	(6) 23.33 ± 0.8ab	(5) 43.6 ± 0.51ab
Adult	(5) 21.2 ± 0.2ab	(5) 42.2 ± 0.2ab
*P*	0.011	0.005

**Fig. 2.**
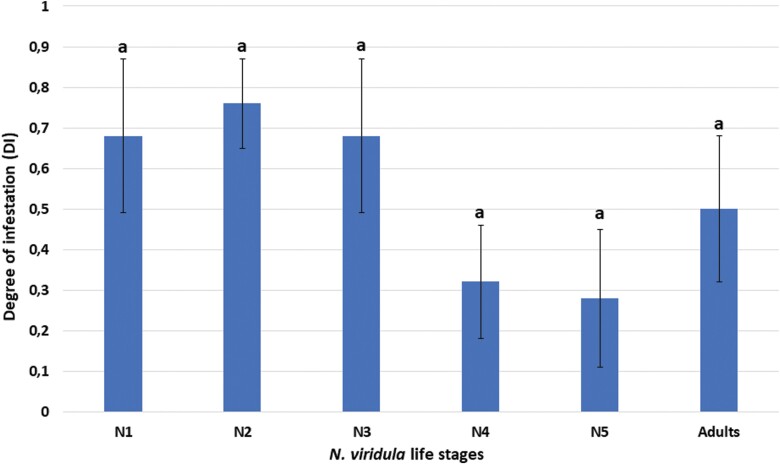
Effects of host stage at parasitization on the parasitoid development in the system *Nezara viridula*–*Aridelus rufotestaceus*: “Degree of infestation” (DI) (=proportion of hosts that were killed by the parasitoid, see the text for explanations). The columns indicate the mean values ± SE. Number of replicates = 5 per stage, each comprising 5 individuals. Different letters above the columns indicate differences in the DI as determined by Median test.

No significant difference was found among host stages for the DI (χ^2^ = 6.67; df = 5; *P* = 0.25). This parameter was, however, lower for N4, N5, and adults (Fig. 2).

### Influence of Temperature

The Log-linear analysis showed that the number of parasitoid cocoons was significantly (χ^2^ = 7.55; df = 1; *P* = 0.006) higher at 25 ± 1 °C (mean ± SE= 2.60 ± 0.60) than at 28 ± 1 °C (=0.60 ± 0.23). The number of parasitoid adults was 2.20 ± 0.45 at 25 ± 1 °C and 0.2 ± 0.19 at 28 °C (at this temperature, only 1 adult was obtained, in 1 replicate). The difference was significant (χ^2^ = 8.63; df = 1; *P* = 0.003). For the unexposed stink bugs, the number of dead individuals was higher (3.4 ± 0.48) at 28 ± 1 °C than at 25 ± 1 °C (=2.4 ± 0.63), but the difference was not significant (χ^2^ = 1.71; df = 1; *P* = 0.192). At both temperatures, all exposed stink bugs died.

No significant difference was found for the parasitoid development times from egg to cocoon between the 2 temperatures, as determined by median test (χ^2^ = 2.49; df = 1; *P* = 0.11). The development times were 22.92 ± 0.52 days at 25 ± 1 °C and 21.4 ± 0.60 days at 28 ± 1 °C. Since at 28 ± 1 °C only 1 adult parasitoid was obtained, no statistical analysis was possible, for the development time from egg to emergence, between the 2 temperatures. This time was shorter at 28 ± 1 °C than at 25 ± 1 °C (=39 vs. 42.05 ± 0.57 days).

### Acceptance and Suitability of *H. halys*

Parasitoid females were observed while attempting to oviposit in second instar *H. halys* nymphs. However, no cocoons were obtained (whereas 3.20 ± 0.54 was the number of cocoons obtained from exposed *N. viridula* nymphs). The number of dead *H. halys* was higher in the exposed (=1.40 ± 0.48) than in the unexposed nymphs (=0.80 ± 0.46), but the difference was not significant (χ^2^ = 0.77; df = 1; *P* = 0.380, Log-linear analysis). Instead, the number of dead individuals in the exposed nymphs was significantly higher for *N. viridula* (=4.00 ± 0.48) than for *H. halys* (=1.40 ± 0.48) (χ^2^ = 11.71; df = 1; *P* < 0.001, Log-linear analysis). No significant difference was found between the numbers of dead individuals in the unexposed *H. halys* (=0.80 ± 0.46) and *N. viridula* (=1.00 ± 0.29) (χ^2^ = 0.09; df = 1; *P* = 0.760).

### Anatomical Studies

**Fig. 3.**
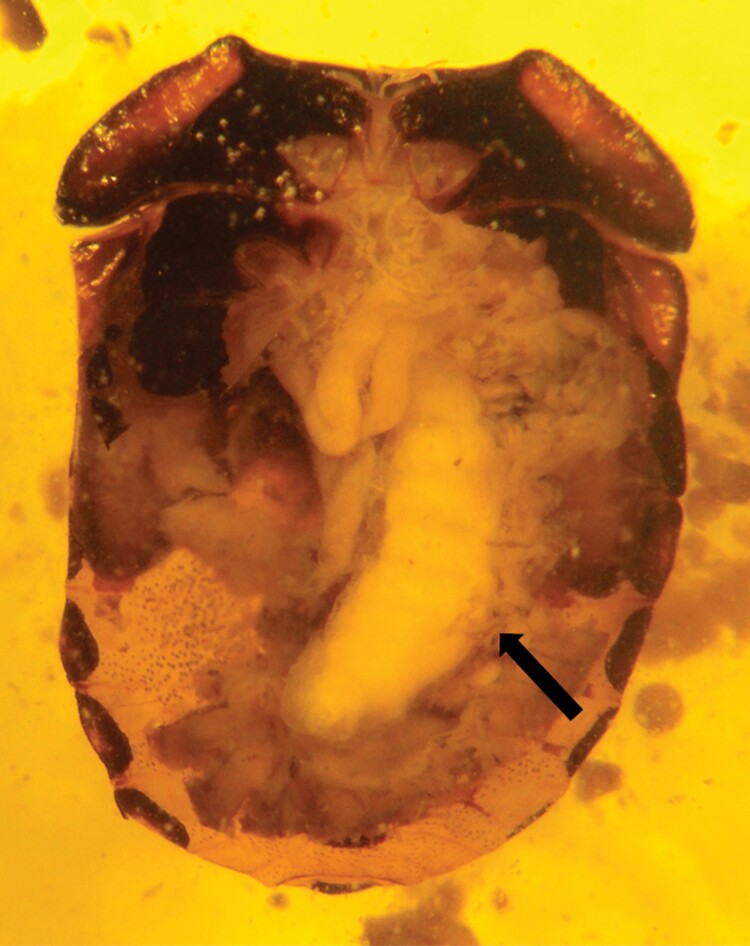
Third instar larva of *Aridelus rufotestaceus* (indicated by the arrow) dead before emergence from the host *Nezara viridula*.

Twenty-nine *N. viridula* and 32 *H. halys* nymphs underwent dissection. In 2 nymphs of *N. viridula*, emergence holes produced by *A. rufotestaceus* larvae were detected. Both parasitoid larvae pupated, but only one reached the adult stage. Head capsules of first instar parasitoid larvae were found in 2 other nymphs, while third instars were detected in further 2 nymphs (Fig. 3), a clear sign of partial development of the parasitoid, which died with the host. Finally, in 23 *N. viridula* nymphs no signs of parasitization were found.

**Fig. 4.**
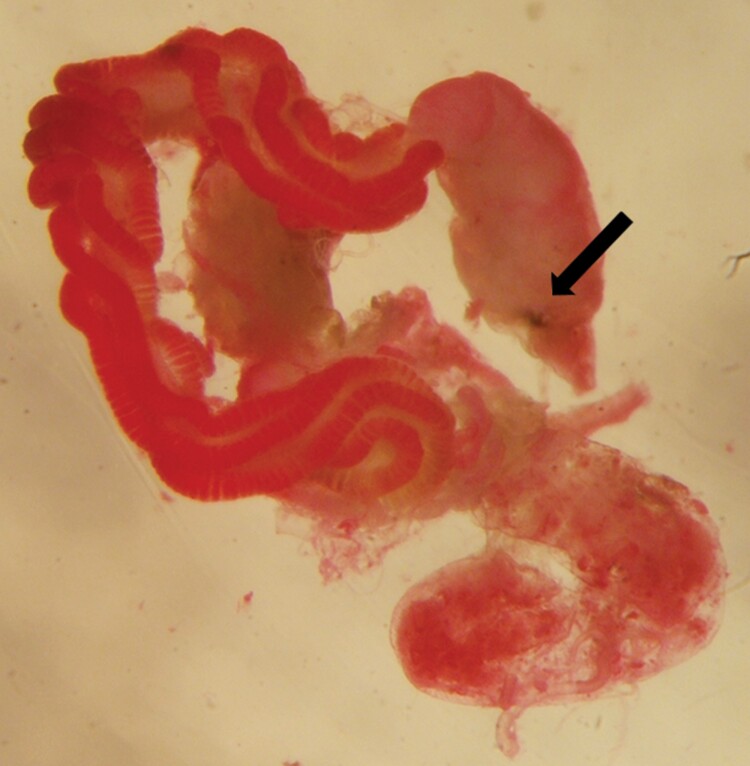
Intestinal lesion (indicated by the arrow) detected in the alimentary canal of a third instar nymph of *Halyomorpha halys* exposed to *Aridelus rufotestaceus*.

No parasitoid emerged from exposed *H. halys* nymphs and no parasitoid head capsule or larva was detected in any of the dissected individuals. A generalized melanization of the alimentary canal and intestinal lesions (Fig. 4) were observed in 12 nymphs. No evidence of oviposition was found in 20 *H. halys* nymphs where all internal organs were unharmed.

## Discussion

One goal of this study was to determine the best stages at parasitization of *N. viridula* for the development of *A. rufotestaceus*. All mobile host stages were exposed to parasitoid females and proved suitable (to a greater or lesser extent) for parasitoid development, including the first instar nymph, which was never tested before (Shaw et al. 2001, Ruberson et al. 2009). Based on the parasitoid cocoon yields, with a difference close to 0.05 significance level, the most suitable host stage for parasitoid development was the second instar nymph, followed by the third and the first instar. The results reported here partially confirm those of Shaw et al. (2001) in that the third instar is the most suitable host stage for *A. rufotestaceus.* These authors used a 24 h parasitoid exposure time compared to a 1 h exposure time used in this study. This exposure time difference may explain why N3 was found to be the preferred stage as opposed to N2 in this study. Despite lack of significance, the DI trend (measuring the proportion of hosts that were killed by the parasitoid [Chabert et al. 2012]) also reflected the preference of *A. rufotestaceus* for the second instar nymphs, followed by the third and the first. The lower survival (and, consequently, the higher DI) of exposed juvenile stages could be related to a lower defense capability shown by early host stages than by advanced ones, as found for other hymenopteran parasitoids of Hemiptera (Mellini 1986, He et al. 2011). It should be noted that, independent of stage at parasitization, the hosts survived 1–2 days after the emergence of the parasitoid larvae, which is consistent with other koinobiont parasitoids, such as *D. coccinellae* (Dindo et al. 2016). The parasitoid adult yields reflected the cocoon yields, suggesting that emergence was not influenced by host stage at parasitization. This is in line with other studies on hymenopteran and tachinid parasitoids, which showed that, once parasitoids successfully pupate, most adults emerge, independently of experimental conditions (Dindo et al. 2016, 2021). The parasitoid total development times varied between 42 and 45.5 days and were in line with those observed, at similar temperatures, by Shaw et al. (2001) and Ruberson et al. (2009). The shortest times occurred in third instar nymphs, further proving that this host stage is also suitable for the rearing of *A. rufotestaceus*, although lower parasitoid cocoon and adult yields were obtained than in the second instar nymphs.

Fewer parasitoid cocoons were obtained at 28 °C than at 25 °C, suggesting that the development of *A. rufotestaceus* was adversely affected by the higher temperature. At 28 °C only 1 parasitoid emerged as an adult and developed more quickly compared with the parasitoids reared at 25 °C. These data do not support 28 °C to be a suitable rearing temperature for *A. rufotestaceus*. Based on the number of adults found, the suggested temperature for rearing this parasitoid is, therefore, 25 °C, which has also proven to be the best temperature for rearing *N. viridula* in both the present and previous studies (Ali and Ewiess 1977, Fortes et al. 2006, Francati et al. 2019). The higher temperature accelerated *A. rufotestaceus* development but resulted in a dramatic drop in parasitoid production. These findings were in line with those shown by Ruberson et al. (2009), that obtained only 1 *A. rufotestacues* adult from *N. viridula* second instar nymphs at 30 °C. Further trials may be performed using different nymphal stages (i.e., the third) as hosts.

During the anatomical observations on exposed *N. viridula* encapsulated *A. rufotestaceus* larvae were never found. Seemingly, the parasitoid larvae could escape the host immune system. The study showed that the trophic activity of *A. rufotestaceus* third instar larva was carried out only on the host’s fat body, whereas no damage was found on other tissues or organs, as also shown for some hymenopteran parasitoids of coccinellids and lepidopterans (Triltsch 1996, Quicke 2015). In the case of *H. halys*, female parasitoids were observed while attempting to oviposit, but no cocoon was obtained, and no larva and no head capsule were detected during the anatomical observations. About one third of the individuals showed a generalized melanization of the alimentary canal and intestinal lesions which might be attributable to the host’s immune response due to hemocytes (plasmatocytes) and/or to the ovipositor action of parasitoid females (Carton et al. 2008). Moreover, in the acceptance and suitability experiment, despite lack of significance, *H. halys* mortality was higher in the exposed than in the unexposed individuals, possibly due to parasitoid activity. More studies are needed to investigate the possibility that at least some *H. halys* nymphs may be accepted by *A. rufotestaceus.*

Based on the results obtained, the mass production of *A. rufotestaceus* for use in augmentative biological control plans against *N. viridula* or other stink bugs is not feasible. The major obstacle is the very long development time which seems difficult to reduce (certainly not through temperature increase). *Aridelus rufotestaceus* may, however, play a useful role in the control of *N. viridula* in a context of conservation biological control, also because it can parasitize all the mobile stages of the target insect pest. It may, therefore, be worth rearing this parasitoid on a small scale, to use it as model nontarget species in studies aimed at testing the side effects of agrochemicals (Marchetti et al. 2009), as it is currently done in our laboratory (research in progress).
